# Anti-neuronal and anti-mitochondrial autoantibodies are associated with lower functional status and more severe respiratory symptoms in post COVID syndrome

**DOI:** 10.3389/fimmu.2025.1642250

**Published:** 2025-09-09

**Authors:** Antje Vogelgesang, Anke Steinmetz, Angela Stufano, Valentina Schino, Domenico Plantone, Agnes Flöel, Guglielmo Lucchese

**Affiliations:** ^1^ Department of Neurology, Universitätsmedizin Greifswald, Greifswald, Germany; ^2^ Physical and Rehabilitation Medicine, Center for Orthopedics, Trauma Surgery and Rehabilitation Medicine, Universitätsmedizin Greifswald, Greifswald, Germany; ^3^ Interdisciplinary Department of Medicine, University of Bari Aldo Moro, Bari, Italy; ^4^ Department of Medicine, Surgery and Neuroscience, University of Siena, Siena, Italy; ^5^ German Centre for Neurodegenerative Diseases (DZNE) Standort Greifswald, Greifswald, Germany; ^6^ Department of Experimental Medicine, University of Salento, Lecce, Italy

**Keywords:** post COVID, autoimmunity, mitochondria, autoantibodies, brainstem

## Abstract

**Introduction:**

We previously identified IgG autoantibodies targeting epitopes within brainstem proteins—disabled homolog 1 (*DAB1*), apoptosis-inducing factor 1 (*AIFM1*), and surfeit locus protein 1 (*SURF1*)—as markers of severe acute COVID-19. This study investigates whether the same autoantibodies contribute to the pathophysiology of Post COVID Syndrome (PCS).

**Methods:**

Using a multiplexed bead-based immunoassay, we measured IgG levels against 18 synthetic peptides derived from *DAB1*, *AIFM1*, and *SURF1* in serum samples from 45 PCS patients and 30 post-COVID controls without long-term symptoms. We employed generalized linear mixed models (GLMM) and nonlinear principal component analysis (CATPCA) to explore associations between antibody levels and clinical variables, including functional status (PCFS), respiratory symptoms, fatigue, cognitive impairment (as assessed by the Montreal Cognitive Assessment, MoCA), and mood.

**Results:**

Higher IgG levels against the three autoantigens significantly predicted PCS at 3 months postinfection (t=2.21, p=0.03), whereas antibodies against a control peptide (polio) showed no such association. CATPCA identified a principal component capturing respiratory symptoms and functional impairment (PCFS), which was also significantly predicted by autoantibody levels (t=2.04, p=0.04). MoCA scores did not correlate with autoantibody levels, and subjective cognitive complaints were paradoxically linked to lower antibody titers and fewer physical symptoms.

**Conclusions:**

The findings from the present explorative study, although largely correlative, appear to suggest a sustained autoimmune response targeting neuronal and mitochondrial proteins in PCS, particularly associated with respiratory dysfunction and reduced functional capacity. The results also highlight potential limitations of standard cognitive screening tools like the MoCA in detecting subtle deficits in PCS. The identified autoantibodies may serve as biomarkers for persistent post-COVID disability. Future research replicating present results on larger samples and specifically investigating a causal link between occurrence of the Auto-Abs and PCS is needed for shaping future immunomodulatory therapeutic strategies.

## Introduction

The COVID-19 pandemic, caused by the SARS-CoV-2 virus, has had a profound impact on global health, leading to long-lasting respiratory, neurological, and immunological complications (1, 2). While significant progress has been made in understanding, preventing, and managing acute manifestations of the disease, the long-term sequelae lasting longer than 3 months after acute disease, defined as post-COVID syndrome (PCS), remain a pressing challenge. PCS encompasses a wide range of symptoms, including persistence of respiratory dysfunction, prolonged fatigue, and cognitive deficits, and thus significantly impairs the quality of life of affected individuals (3, 4). Emerging research also suggests potential long-term neurodegenerative consequences and biomarkers of neuronal damage are elevated in both acute and chronic phase of the disease even in absence of neurological symptoms (5–7). Currently, it appears that aberrant immune responses are among the main mechanisms contributing to neuronal damage and long-term consequences of COVID-19 (5).

Autoimmune responses, characterized by the production of autoantibodies (auto-Abs), have been implicated in the pathophysiology of both acute and long-term COVID manifestations (8, 9). Previous studies have highlighted the role of auto-Abs against various neuronal antigens in the context of acute severe COVID (10, 11). Focusing specifically on PCS, a recent review examined the potential association between auto-Abs and persistent symptoms following COVID. The authors concluded that the occurrence of auto-Abs might play a role in PCS, even though the reviewed studies did not provide definitive evidence (12). Nevertheless, subsequent research further corroborated the role of auto-Abs in PCS. First, a study investigated new-onset auto-Abs, as opposed to well-established ones, in patients suffering from PCS and showed that novel auto-Abs correlated with disease severity in the acute phase (13). A subset of the Abs was found in the CSF and was associated with neuropsychiatric symptoms of PCS. Moreover, the study suggested molecular mimicry and epitope spreading as plausible mechanisms for the occurrence of different subsets of new-onset auto-Abs. However, epitope spreading following tissue damage during acute COVID may lead to the production of novel autoantibodies as an epiphenomenon, without necessarily implying their pathogenicity (14, 15). Evidence supporting a pathogenic role of autoantibodies in PCS arises from a series of recent studies that demonstrate a causal link between novel auto-Abs and PCS. Indeed, two different research groups showed that the transfer of anti-neuronal IgG isolated from Long COVID patients into mice could induce PCS symptoms (16). In addition, therapeutic techniques for removing circulating Abs, such as plasma exchange and, more specifically, immunoadsorption, proved to be effective in treating PCS (17, 18). In sum, it appears that an array of novel auto-Abs is involved in the pathogenesis of PCS and some of them might be induced by molecular mimicry between the virus and human neuronal antigens.

We previously identified novel auto-Abs against brainstem neuronal antigens, such as *DAB1*, *AIFM1*, and *SURF1*, which share epitopes with SARS-CoV-2 and are linked to autonomic modulation of respiratory function, highlighting a potential mechanism underlying the severe respiratory and neurological symptoms observed in acute COVID (**Table 1**) (11). These auto-Abs, specifically of the IgG isotype, were associated with an anti-viral immune response and clinical severity, suggesting their involvement in the pathogenesis of the disease. The involvement of the human proteins *DAB1*, *AIFM1*, and *SURF1* in acute COVID provides a basis to investigate autoimmunity against the same antigens also in PCS. Given a possible overlap in autoimmune responses between acute severe COVID and PCS, there is a critical need to explore the role of these auto-Abs in the context of long-term sequelae. Persistent disability as observed in PCS patients suggests an ongoing residual autoimmune activity that may contribute to the pathophysiology of long-term complications affecting respiratory function by damaging brainstem respiratory centers, as previously shown in acute COVID (11).

**Table 1:** Peptide sequence sharing between brainstem and mitchondrial autoantigens and SARS-CoV-2.

Predicted epitope	Human brainstem protein (epitope position) [UniProt-ID; Gene]	SARS-CoV2 protein (epitope position) [UniProt-ID; Gene]
GSQASS	Disabled homolog 1 (560-565) O75553; *DAB1*	Nucleoprotein (179-184) P0DTC9; N
LNEVAK	Apoptosis-inducing factor 1, mitochondrial (601-606) O95831; *AIFM1*	Spike glycoprotein (1186-1191, S2-domain) P0DTC2; S
SAAEAS	Surfeit locus protein 1 (47-52) Q15526; *SURF1*	Nucleoprotein (250-255) P0DTC9; N

The provided data includes information on hexapeptides, along with their associated human and viral proteins. These hexapeptides have been previously identified as epitopes targeted by cross-reactive antibodies during the immune response to the virus (8). The information encompasses variants of concern, such as B.1.617.2 (Protein Data Bank ID: 7W92, https://www.rcsb.org/structure/7W92) and B.1.1.529 (Protein Data Bank ID: 7QO7; https://www.rcsb.org/structure/7QO7). Adapted from Ref. (57).

Therefore, the present study aimed to investigate the occurrence and pathogenic role of auto-Abs against *DAB1*, *AIFM1*, and *SURF1* in patients suffering from PCS. We additionally explored the association of these auto-Abs with various clinical manifestations of PCS, including fatigue, respiratory symptoms, cognitive deficits (as both self-reported symptoms and scale-based), and psychological aspects, as well as overall functional status (as measured by the Post COVID-19 Functional Status Scale, PCFS). By extending our understanding from acute severe cases to long-term manifestations, novel insights may be gained into the mechanisms underlying PCS and potential therapeutic targets to ameliorate its consequences.

## Methods

### Patients and samples

Serum samples were obtained from 45 patients with PCS after acute SARS-CoV-2 infection confirmed via PCR-Test as well as 30 patients who were affected by acute COVID without long-lasting sequelae. Sample size was estimated by way of a Montecarlo simulation power analysis assuming a medium size effect corresponding to a OR of ca 3.5. The included PCS patients were part of the Greifswald PoCoRe cohort and fulfilled the WHO criteria for post-COVID; continuing or new symptoms 3 months after a PCR-confirmed COVID-19 infection lasting for at least 3 months without any other explanation (19). Sera from PCS patients were sampled at 3 (T1) and 6 (T2) months after acute COVID and stored at the institutional biobank (20). Sera from patients without PCS were sampled at 3 months only.

Patients with PCS presenting at a university outpatient rehabilitation clinic were recruited for the Greifswald PCS Rehabilitation Study and Research (PoCoRe) cohort since April 2021. Included patients were referred mainly by local or regional physicians and were predominately non-hospitalized patients with a mild acute course of PCR-confirmed COVID-19 (21). PCS patients received a wide clinical and functional assessment, including symptom description, assessments of fatigue (Fatigue Assessment Scale, FAS), cognition (Montreal Cognitive Assessment, MoCA), psychological aspects (Patient Health Questionnaire-Depression Module, PHQ-9; General Anxiety Disorder, GAD-7) and function (Post COVID-19 Functional Status Scale, PCFS). In particular, the PCFS is a tool specifically developed to assess the functional status and the impact of PCS on daily activities (22). This scale helps to understand the long-term effects of PCS on individuals who have recovered from the acute phase of the infection. The PCFS is a simple and quick assessment tool that classifies the level of functional impairment into four grades. Grade 0 indicates no impairment. Grade 1 is defined by negligible functional limitations with mild symptoms or limitations that do not interfere significantly with normal activities of daily life. Grade 2 requires slight functional limitations with noticeable symptoms that slightly impact their daily activities. The patient can still perform all essential tasks but with some effort or discomfort. Grade 3 corresponds to moderate functional limitations. Assistance or modifications are needed to perform certain tasks. Grade 4 indicates severe functional limitations requiring substantial assistance or adaptations. Biosamples were also collected. The first 45 patients reaching T2 for which biosamples were available were consecutively included in the present study.

Controls were prospectively included between January and June 2022 at the University Hospital of Bari during the occupational health surveillance performed according to the internal procedures for SARS-CoV-2 positive employees. The clinical characteristics of the participating patients can be found in **Table 2**. Diagnosis of previous SARS-CoV2 infection was made through an oropharyngeal swab with subsequent rt-PCR analysis. The real-time RT-PCR assay was performed in a laboratory accredited by the Local Health Authority, targeting SARS-CoV-2 envelope (E), replicase (RdRP), and nucleocapsid (N) genes, according to the WHO protocol (23). Briefly, nasopharyngeal (NP) or oronasal (OP) swabs were subjected to nucleic acid extraction with the MagNA Pure System (Roche Diagnostics), in accordance with the manufacturer’s instructions. The presence of the SARS-CoV-2 genes was evaluated by a commercial real-time RT-PCR assay (Allplex 2019-nCoV Assay; Seegene). Amplification and detection were performed for 45 cycles on a BioRad CFX96 thermocycler (BioRad Laboratories, the Netherlands). Samples were considered positive at molecular screening if all three E, N, and RdRP genes were detected.

**Table 2:** General characteristics of the study population.

Variables	Cases (n 45) (T1) N (%) Median (Range)	Cases (n 45) (T2) N (%) Median (Range)	Controls (n 30) N (%) Median (Range)
Sex	35 F (78) 10 M (22)	35 F (78) 10 M (22)	10 F (33) 20 M (67)
Age (years)	51 (20-82)	51 (20-82)	54 (29-68)
Days from SARS-CoV-2 infection	119 (49- 176)	233 (143- 307)	143 (109-249)
Symptoms			
- Cognition	21 (46.7)	21 (46.7)	0 (0.0)
- Headache	6 (13.3)	6 (13.3)	0 (0.0)
- Respiratory	13 (28.9)	13 (28.9)	0 (0.0)
- Fatigue	31 (68.9)	31 (68.9)	0 (0.0)
- Gastroenteric	2 (4.4)	2 (4.4)	0 (0.0)
N. symptoms			
- One	22 (48.9)	22 (48.9)	0 (0.0)
- Two	18 (40)	18 (40)	0 (0.0)
- Three	5 (11.1)	5 (11.1)	0 (0.0)
PCFS	3 (1-4)	3 (2-4)	0 (0-1)
PHQ-9*	1 (1-2)	1 (1-2)	0 (0-1)
GAD-7	6 (1-20)	8 (0-21)	–
FAS	30 (16-49)	33 (10-50)	–
MoCA	26 (15-30)	26 (19-30)	–
Steroid treatment for acute COVID	0 (0)	0 (0)	6 (20)
At least 1 COVID-Vaccine dosis	26 (58)	26 (58)	100 (100)
Comorbidities			
- Hypertension	16 (35.6)		6 (20)
- Diabetes	4 (8.9)		1 (3.3)
- Other Cardiovascular	4 (8.9)		1 (3.3)
- Dysthyroidism	6 (13.3)		0
- COPD	2 (4.4)		1 (3.3)
-Asthma	2 (4.4)		0
- Arthrosis	3 (6.7)		0
- Arthritis	5 (11.1)		0
- Allergy	2 (4.4)		0
- Gastroenteric	4 (8.9)		0
- Neurologic	7 (15.6)		1 (3.3)
- Depression	2 (4.4)		0

The table shows baseline information for 45 patients affected by post-COVID syndrome (PCS) as well as 30 control patients who fully recovered from acute COVID-19. Characteristics include age, sex, number and type of PCS symptoms reported, days between confirmed COVID-19 and timempoint of PCS assessment, treatment and vaccination status for COVID as well as comorbidities at the time of PCS assessment. Scores on the following scales were assessed: Fatigue Assessment Scale, FAS; Montreal Cognitive Assessment, MoCA; Patient Health Questionnaire-Depression Module, PHQ-9; General Anxiety Disorder, GAD-7; Post COVID-19 Functional Status Scale, PCFS. PHQ-9 scores were classified as follow: scores 5–9 as suggestive of light depressive symptoms = 1; scores 10–14 as suggestive of moderate depressive symptoms = 2; scores 15–27 suggestive of severe depressive symptoms = 3. PCS assessment and sera collection occurred at 3 (T1) and 6 (T2) months after acute COVID from post-covid-syndrome (PCS) patients, sera from controls (patients without PCS) were sampled at 3 months only.

Both PCS patients and controls were interviewed face-to-face by trained physicians using a structured questionnaire. Information was collected on demographic characteristics, smoking and alcohol habits, regular medication use, symptoms during acute COVID-19, and treatments received. Participants were also actively questioned about any new, persistent, or worsened symptoms following COVID-19, based on the symptom list provided by Subramanian et al. (24) The diagnosis of PCS was assigned or excluded in accordance with the WHO definition (25), including in the control group only individuals who did not report the persistence or development of any symptoms consistent with PCS, whether intermittent or continuous.

In addition to the clinical interview, controls completed the Post COVID-19 Functional Status Scale (PCFS) and the Patient Health Questionnaire-9 (PHQ-9). These instruments were used to screen for functional limitations and depressive symptoms potentially related to post-COVID-19 sequelae. Controls did not complete the additional questionnaires administered to PCS patients (Fatigue Assessment Scale, Montreal Cognitive Assessment, Generalized Anxiety Disorder-7) due to their healthy status and absence of persistent symptoms. On the day of questionnaire administration, venous blood samples (10 mL) were collected in two separate Vacutainer tubes containing EDTA and a separating gel, respectively. Samples were stored at -80 °C until analyses and were heat-inactivated at 56 °C for 30 min immediately before run in immunoassays.

The study protocol was approved by the ethics committee of the University Medicine Greifswald (No. BB 053/21) and the University Hospital Bari (No. 6663, 2021), and was conducted in accordance with the Helsinki Declaration. All participants provided written informed consent.

### Multiplexed immunoassay

A multiplexed bead-based lumina immunoassay was used to assess a binding of auto-Abs from the patient sera to 18 custom peptides and polio peptide KEVPALTAVETGATGG as positive control. Peptides were synthesized with a C-terminal eBio-tag. The eBio-tag is a biotin unit coupled via an ethylene glycol linker to the side chain of glutamic acid. The 18 peptides were selected because they had previously shown higher binding among a larger set of epitopes from the three autoantigens *DAB1*, *AIFM1*, and *SURF1* that were already shown elsewhere to be targets of autoimmunity in COVID-19 (11) and are listed with the corresponding proteins in the **Supplementary Material**. Coupling of peptides to MAGPIX^®^ MagPlex-Avidin Microspheres (Luminex/DiaSorin) was carried out using 5 µg peptide per 106 beads. For this, the respective amount of beads was transferred to low-binding microcentrifuge tubes and placed in a magnetic separator for 2 minutes. After removing the supernatant, pelleted microspheres were resuspended in coupling buffer (The Blocking Solution, Candor Bioscience) by vortexing and sonication for 20 seconds. The biotinylated peptides were diluted in coupling buffer and subsequently mixed with the respective microsphere suspension. The peptide-microsphere suspension was incubated for 30 minutes at room temperature with mixing by rotation in the dark (coupling reaction). Afterwards, the microcentrifuge tube was placed in a magnetic separator for 2 minutes and the supernatant was removed. The peptide-coupled microspheres were washed three times with storage buffer (The Blocking Solution with 0.02% Tween 20 and 0.05% sodium azide). Finally, the pelleted microspheres were resuspended in storage buffer, adjusted to 106 beads/ml, and stored at 4 °C until use.

Peptide-coupled microspheres were combined to yield a total of 2,500 microspheres per coupled peptide in a volume of 100 µl per single measurement. Subsequently, 100 µl of the mixed microsphere suspension were transferred to 96-well non-binding plates (Greiner Bio-One), washed on a magnetic plate washer using the washing buffer (PBS, pH 7.4 with 0.05% Tween 20 (PBS/T)), and incubated with the sera diluted in assay buffer (Low Cross Buffer (Candor Bioscience) with 0.05% Tween 20 and 2 mg/ml normal goat serum, 1:50) in duplicate for 16 h at 4 °C and shaking at 600 rpm. Binding of serum antibodies was detected with the secondary antibody (Goat anti-human IgG (Fc)-PE (Invitrogen) at 1 µg/ml in PBS/T) and 60 min staining at RT and shaking at 700 rpm followed. The median fluorescence intensity was detected and quantified with the Luminex MAGPIX^®^ system. The average and the deviation of the median fluorescence intensities of duplicate measurements were calculated.

### Statistics

We employed a Generalized Linear Mixed Model (GLMM) to investigate the relationship between the occurrence of IgG against the three auto-antigens and PCS. The first GLMM modeled the occurrence of PCS using the measured fluorescence intensity for the IgG against the peptides from the three neuronal antigens and the interval from acute COVID onset to serum sampling in days and peptide as fixed effects as well as the peptide as a random effect. The Link function was binary logit. For this analysis, the values from the PCS-Patient samples collected at T1 were used. A second GLMM with a binary logit link function was fitted adopting the measured fluorescence intensity for IgG against the polio peptide used as positive control and the interval from acute COVID onset to serum sampling in days as fixed effects. The predicted variable was again the occurrence of PCS.

The relationship between self-reported symptoms, fatigue, cognition, depression, anxiety, and function as assessed with standardized tests, and the fluorescence intensity measured from PCS patient samples at T1 and T2 was investigated with dimensionality reduction methods. Non-linear principal component analysis (NLPCA), also known as categorical component analysis (CATPCA) was used to map the 13 variables reported in **Table 2** (lines 2 to 10) for the 45 PCS patients onto a lower dimensional space thus eliminating eventual redundancy and minimizing multicollinearity. The CATPCA uses optimal scaling quantification to extend linear PCA to ordinal and nominal categorical variables (26). The FAS, MoCA, PHQ-9, GAD-7, and PCFS scores, age at sampling, days between acute disease and sampling, the self-reported occurrence of fatigue, cognitive impairment, headache, respiratory, and gastrointestinal symptoms as well as an overall number of self-reported symptoms were included as original variables in the CATPCA. Solutions were computed for a three to six component range using a non-parametric bootstrap to assess the significance of the loadings on the components. The three-dimension solution was adopted for analysis because all of the confidence intervals of the loadings on the fourth component included the value zero and the first three components explained 48% of the total variance indicating adequate fit.

Finally, we adopted three separate GLMM to predict each of the three series of CATPCA scores, one for each component. Each GLMM was fit with fluorescence intensity averaged across peptides as well as timepoint as fixed effects and subject as random effects thus accounting for the repeated measure design. Additionally, we run one more GLMM to differentiate between T1 and T2 using the measured fluorescence intensity for the IgG against the peptides from the three neuronal antigens, peptide, and interval from acute COVID onset to serum sampling in days as fixed effects as well as subject as a random effect and. The mixed model analyses were performed in the MATLAB programming environment (R2018b; MathWorks, Natick, MA). The CATPCA was carried out in SPSS 29.0 (Armonk, NY: IBM Corp).

## Results

**Figure 1:**
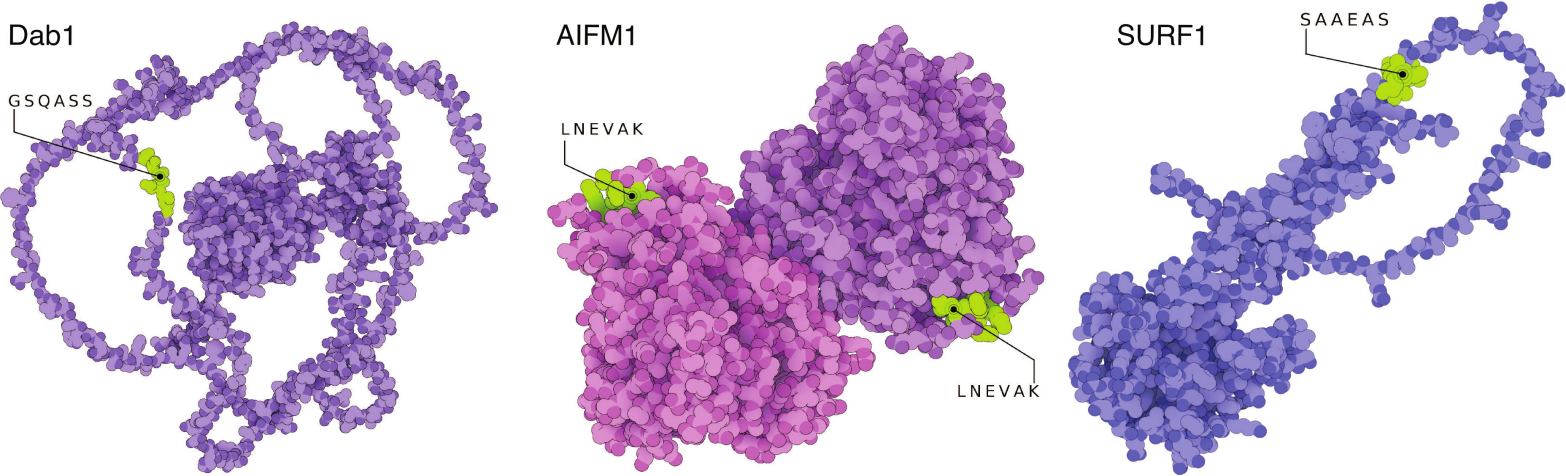
Structural model of the three autoantigens disabled homolog 1 (*DAB1*), apoptosis-inducing factor 1 (*AIFM1*), and surfeit locus protein 1 (*SURF1*). Cristallography and modelled structures show the predicted and confirmed epitopic sites exposed in known conformational states of the three target proteins. The sites are therefore freely accessible to auto-Abs for recognition and binding. *AIFM1* is presented in its dimeric form. The 3D-illustrations were produced with The Protein Imager (58) with cristallography data obtained from Ref. (59) and structure predictions from Ref. (60). Adapted from Ref. (11).

**Figure 2:**
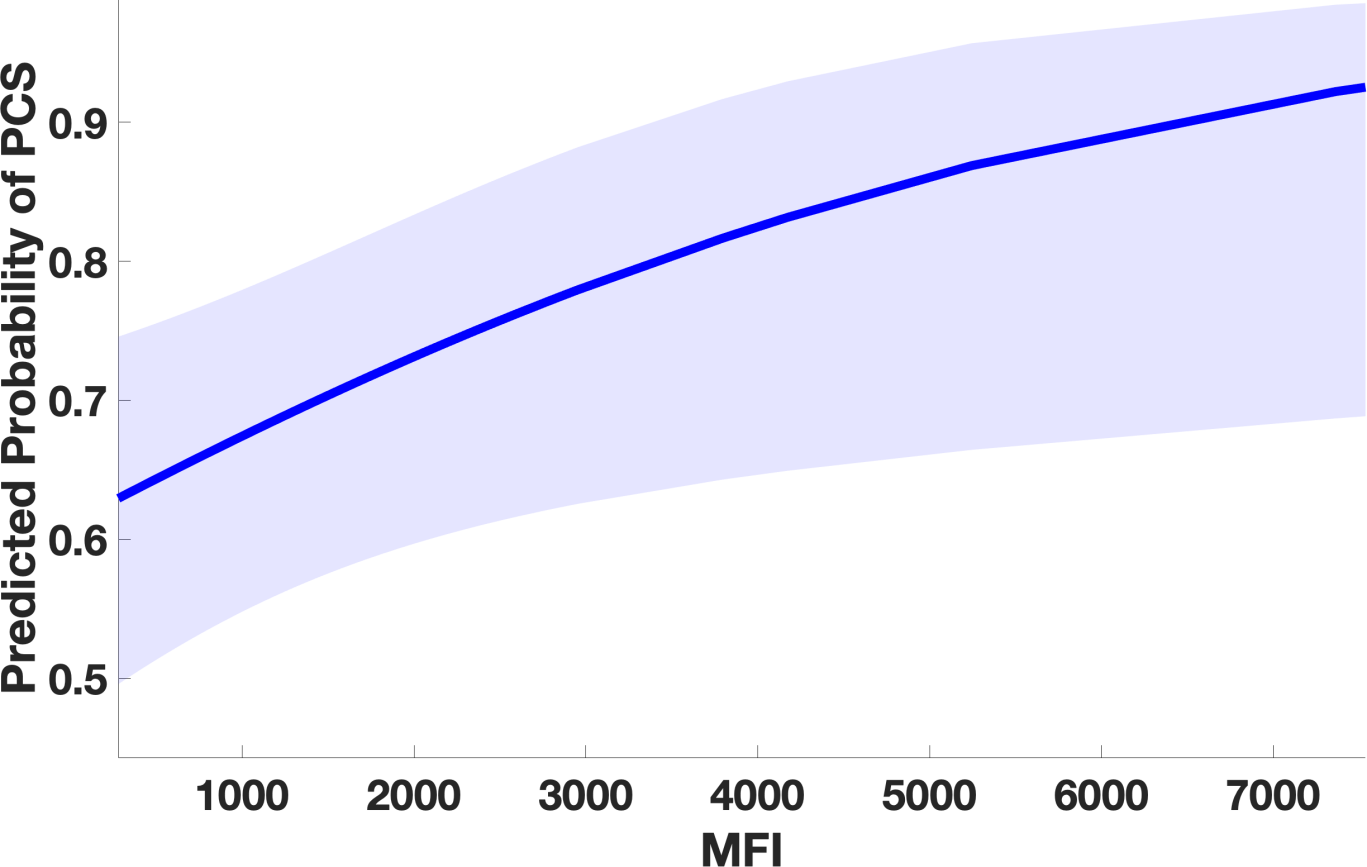
Marginal effect plot from the GLMM showing significant association between higher auto-Ab levels against DAB1, AIFM1, and SURF1 and occurrence of PCS. Higher levels of IgG against the three autoAg, corresponding to higher immunofluorescence levels, significantly predict the probability of PCS occurrence indicating an association between Ab-mediated-autoimmunity against the three proteins and occurrence of long-term sequelae after COVID. The line represents the predicted probability of PCS as a function of measured antibody level. The shaded area in the plot represents the 95% confidence interval around the predicted probability. (PCS=post covid syndrome, MFI=mean fluorescence intensity).

We adopted a GEE to examine the relationship between PCS and the occurrence of IgG against DAB1, AIFM1, and SURF1, comparing sera sampled approximately 12 weeks after infection from patients affected by PCS with patients with no long-term sequelae. Higher measured immunofluorescence intensity significantly predicted the occurrence of PCS at three months (as illustrated in **Figure 1**), indicating an association between long-term symptoms and higher IgG levels against the autoantigens. Specifically, the analysis showed a main effect of immunofluorescence on PCS occurrence (t=2.21, df=1276, p=.03) (**Figure 2**) with no significant peptide effect. No significant effect of IgG against the polio peptide as a predictor of PCS occurrence emerged from the second GLMM.

**Figure 3:**
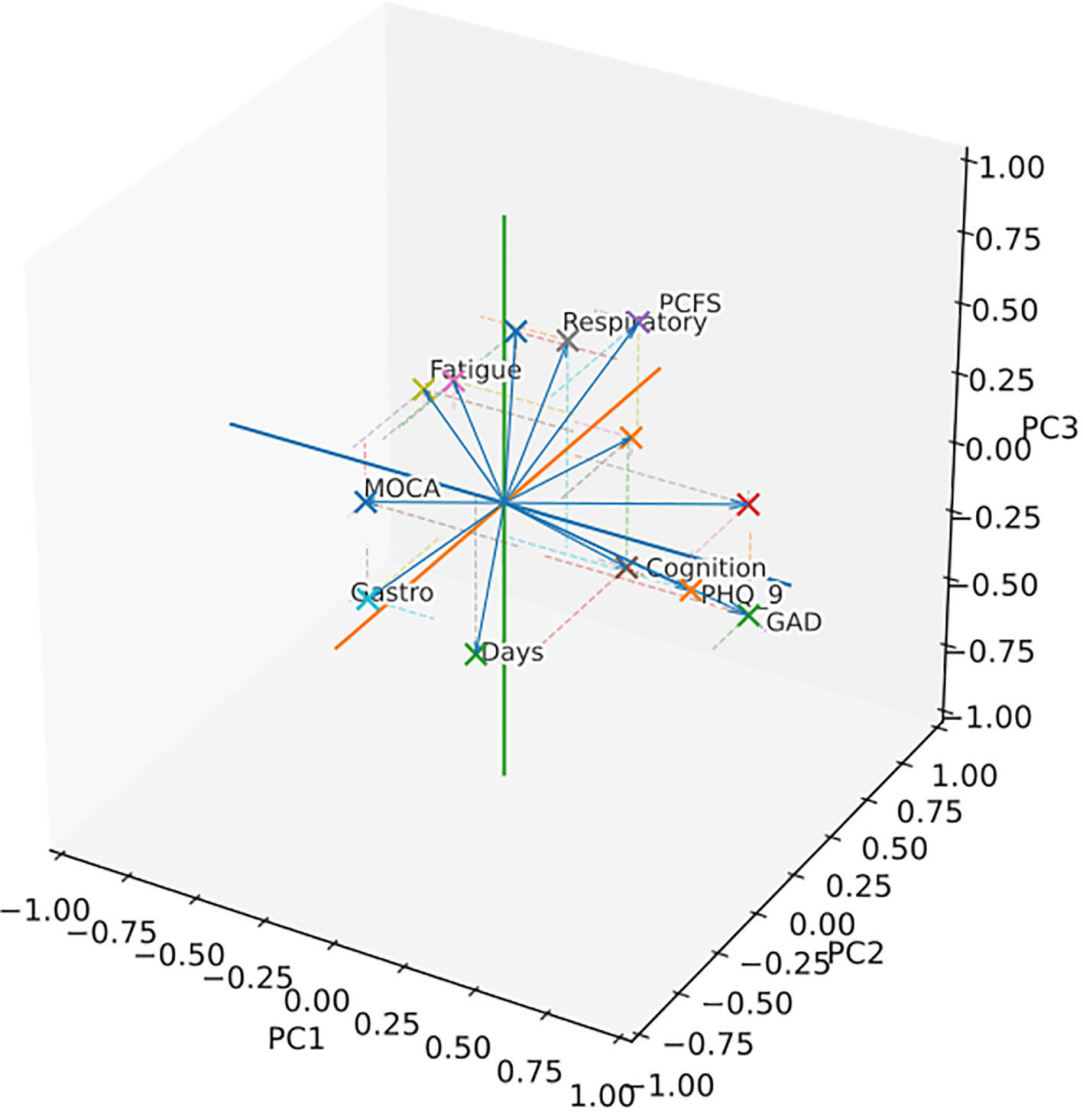
Three-dimensional loading plot from Categorical Principal Components Analysis (CATPCA). Arrows depict the correlations between optimally scaled variables and PCs 1–3; direction indicates sign and length reflects strength. Only variables with |loading| ≥ 0.40 are shown; labels mark the two most positive and two most negative loadings per component (the label for “NSymptoms” is suppressed). In CATPCA, loadings are analogous to conventional PCA: for nominal variables they summarize relationships across categories (with category quantifications detailing contributions), and for ordinal variables they can be interpreted like continuous variables because optimal scaling preserves order. High absolute loadings denote strong influence on a component, whereas values near zero suggest minimal contribution. Abbreviations: FAS, Fatigue Assessment Scale; MoCA, Montreal Cognitive Assessment; PHQ-9, Patient Health Questionnaire-Depression Module; GAD-7, General Anxiety Disorder; PCFS, Post-COVID-19 Functional Status Scale.

**Figure 4:**
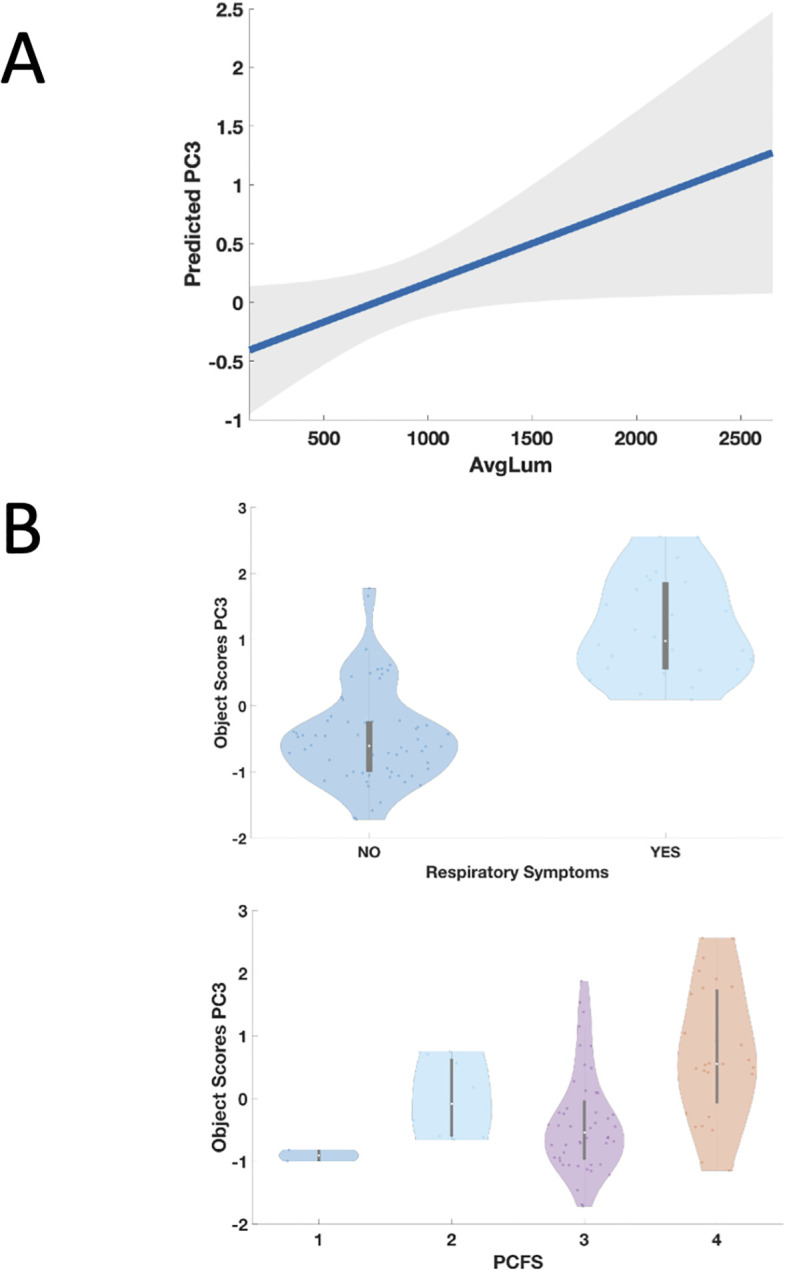
Panel **(A)** Partial regression plot from the GLMM showing significant association between higher auto-Ab levels against DAB1, AIFM1, and SURF1 and the third principal component (PC3). Higher levels of IgG against the three autoAg, corresponding to higher immunofluorescence averaged across the 18 peptides (AvgLum), significantly predict the third principal component (PC3), corresponding to higher frequency of occurrence of respiratory symptoms and to worse functional status as indexed by the PCFS (**Table 3**). The line represents the predicted probability of PC3 as a function of measured antibody level. The shaded area in the plot represents the confidence interval around the predicted probability. Panel **(B)** Relationship between occurrence of respiratory symptoms in PCS patients, Post COVID-19 Functional Status Scale (PCFS) and the third principal component (PC3)**.** Presence of respiratory symptoms (upper panel) and higher scores on the PCFS (lower panel) were associate with higher scores on the PC3 which in turn were significantly predicted by higher auto-Ab levels against DAB1, AIFM1, and SURF1 as described in the Results.

**Figure 5:**
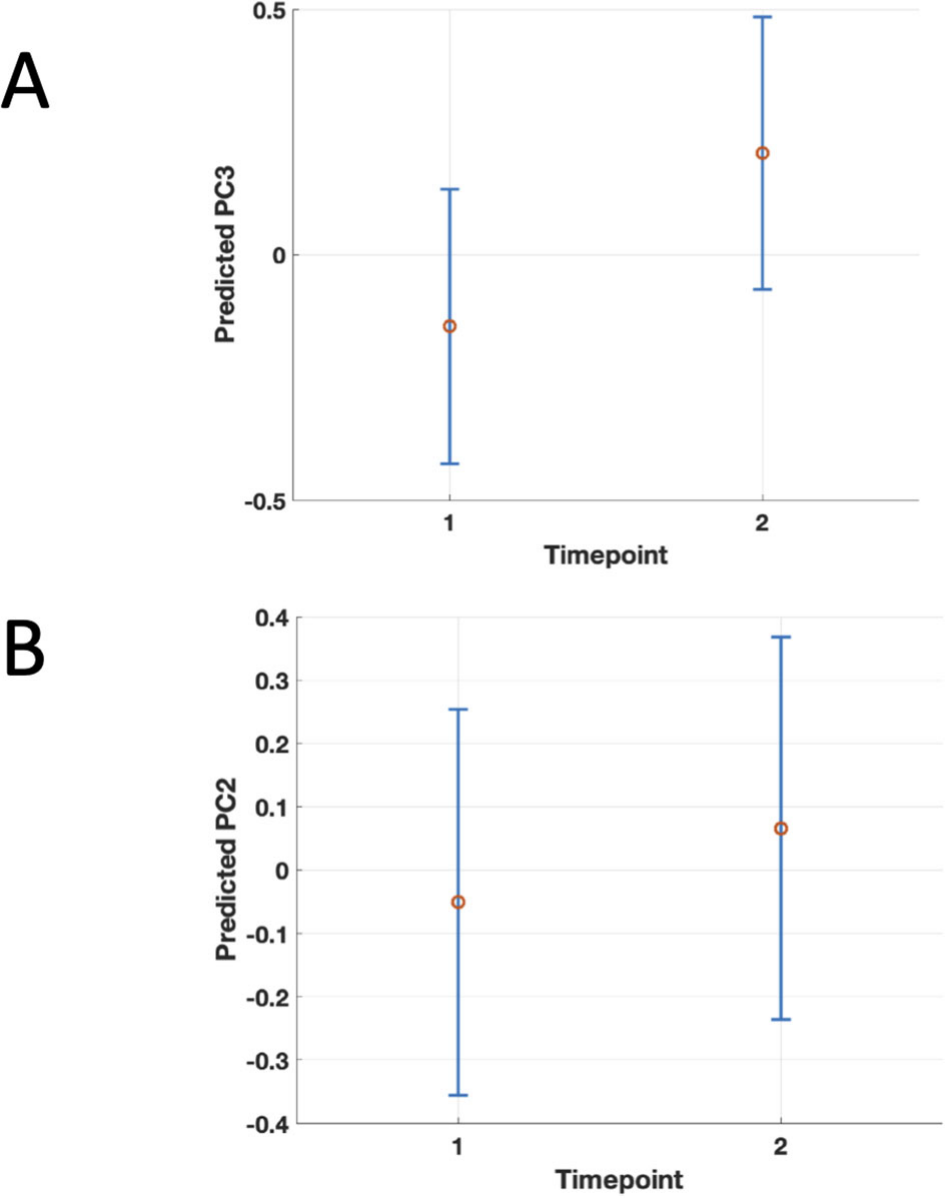
Population-level marginal predicts values of PC3 (Panel A) and PC2 (Panel B) by timepoint from the linear mixed-effects model. Points show the predicted mean at fluorescence intensity fixed fixed to the sample grand mean (i.e., collapsing over subjects); vertical error bars are 95% confidence intervals for the mean prediction. The figure shows the significant association between higher second (PC2) and third principal component (PC3) scores and the second timepoint, 6 months after COVID, suggesting worsening of the corresponding clinical symptoms between 3 and 6 months after acute infection in PCS patients..

A three-dimensional CATPCA was adopted to investigate the correlation structure between symptoms and neuropsychological and psychometric scores in PCS patients. The original variables MoCA, PHQ-9, GAD-7, FAS, occurrence of headache, and fatigue loaded with an absolute coefficient higher than 0.4 on the first component, the one explaining the largest amount of variance in the data (20%). The second component resulted from the combination of FAS, PCFS, occurrence of cognitive symptoms, occurrence of headache, total number of symptoms, and age at sampling, accounting for 16% of the total variance. The third component explained 12% of the variance in the data and was the combination of the occurrence of respiratory symptoms and cognitive symptoms, PCFS, and days between acute COVID and sampling (**Table 3** and **Figure 3**). Three GLMM were calculated using the fluorescent intensity averaged across peptides and time points as predictors of the three principal components. The analysis showed no significant effects on the first (immunofluorescence: t=.85, df=87, p=.39, timepoint t=.82, df=87, p=.41), no significant effect of immunofluorescence on the second (t=-1.5, df=87, p=.15), and a main effect of immunofluorescence (t=2.04, df=87, p=.04) (**Figure 3A**) as well as a main effect of timepoint (t=3.23, df=87, p=.001) on the third principal component. A similar main effect of time on the second component was significant (timepoint t=2.1, df=87, p=.03).Overall, PCFS and occurrence of respiratory symptoms loaded with positive coefficients on the third component, as shown in **Table 3** and **Figure 3**. This indicates a direct correlation with the component for the continuous variables. Therefore, higher immunofluorescence predicted higher scores on the third principal component (**Figure 4A**), which in turn corresponded to worse functional status as indexed by the PCFS and was associated with more frequent self-reported occurrence of respiratory symptoms three months after COVID (**Figure 4B**). These findings indicate that higher levels of IgG targeting the three brain autoantigens *DAB1*, *AIFM1*, and *SURF1* were associated with a higher disease burden in the 6 months following acute infection. Moreover, the timepoints effect on principal component two and three showed higher scores on the components at 6 months as compared to 3 months (**Figure 5**). The GLMM to differentiate between T1 and T2 using the measured fluorescence intensity for the IgG against the peptides from the three neuronal antigens, peptide, and interval from acute COVID onset to serum sampling in days as fixed effects as well as subject as a random effect and returned a significant effect only for the interval in days (p<.001).

**Table 3:** Original variables with a Loading >.40 on the three principal component resulting from the Categorical Principal Components Analysis (CATPCA). IN CATPCA *loadings* represent the correlations between optimally scaled variables and the extracted principal components.

PC1		PC2		PC3	
GAD-7	.741	Neurological Symptoms	.737	Respiratory Symptoms	.724
PHQ-9	.645	Cognition	.601	PCFS	.417
FAS	.622	PCFS	.566	Cognition	-.510
Headache	-.439	FAS	.446	Days between acute disease and sampling	-.587
Fatigue	-.548	Age	.444		
MoCA	-.570	Headache	.439		
		Fatigue	.436		
		Gastroenterological Symptoms	-.430		

They indicate both the strength and direction of a variable’s association with a component, analogous to loadings in conventional PCA. For nominal variables, loadings summarize the relationship across all categories, while category quantifications provide detailed contributions. For ordinal variables, loadings can be interpreted similarly to continuous variables, as the optimal scaling preserves order. High absolute loadings denote strong influence on a component, whereas values near zero suggest minimal contribution. (FAS, Fatigue Assessment Scale; MoCA, Montreal Cognitive Assessment; PHQ-9, Patient Health Questionnaire-Depression Module; GAD-7, General Anxiety Disorder; Post COVID-19 Functional Status Scale).

## Discussion

In our study, we observed that autoantibody (auto-Ab) responses against neuronal proteins *DAB1*, *AIFM1*, and *SURF1* were a significant predictor of PCS following acute disease. Our findings suggest a sustained autoimmune response in PCS, contrasting with transient autoimmunity in acute COVID phases without long-term sequelae.

When exploring the association of auto-Ab against the three brainstem Ag and psychometric, neuropsychological, and clinical manifestations of the disease, we first conducted a non-linear principal component analysis (NLPCA) to avoid redundancy, multicollinearity, and to minimize multiple comparisons. This led to three principal components corresponding to as many main clusters of original variables. Interestingly, all the cognitive and psychometric test results (MoCA; PHQ-9; GAD-7) cluster together with subjectively reported Fatigue and Fatigue Assessment Scale (FAS) scores in the first component, indicating a high interrelation between these measures and possibly common underlying causes and mechanisms. The second cluster of variables includes subjectively reported cognitive deficits, headache, FAS, and Post COVID-19 Functional Status Scale (PCFS) scores. This second cluster suggests independence between cognition as assessed neuropsychologically, specifically with the MoCA, and as subjectively reported because the two types of data cluster in different components. This notion is coherent with previous research showing that self-reported cognitive complaints in COVID-19 are unrelated to actual cognitive performance (27, 28). Finally, the third cluster of variables identified by the principal component analysis contains again PCFS scores and self-reported cognitive deficits with the addition of the self-reported occurrence of respiratory symptoms and time in days occurring between acute COVID and sampling (**Table 3**). Overall, these results of the exploratory CATPCA suggest that reduced functional status in PCS as captured by the PCFS might result from the combined effect of various clinical and pathophysiological domains in PCS. On the one hand, fatigue and cognitive deficits seem to be tightly linked and cluster together with PCFS scores in the second component. On the other hand, the third component suggested a commonality between respiratory symptoms and PCFS-measured disability.

Moreover, scores on this third component were significantly predicted by auto-Ab levels in a regression model indicating that higher levels of Ab against the three proteins were linked to persisting respiratory symptoms and overall worse functional status as indexed by the PCFS. Our results indicate that the antibody-based autoimmune response against the three proteins not only characterizes PCS patients compared to patients that have recovered without long-term sequelae from acute COVID but is also associated with higher PCFS scores and therefore higher functional limitations and disability in PCS patients.

The findings in this study are consistent with the biological functions of the investigated autoantigens. As previously discussed elsewhere, *DAB1*, *AIFM1*, and *SURF1* are linked to autonomic modulation of respiratory function in the brainstem and are associated with more severe disease in the acute phase of COVID (11). The persistence of autoantibodies against the three neuronal proteins in PCS underscores their potential role in ongoing respiratory symptoms and persistent disability in PCS. A further mechanism linking PCS to functional status and disability, as suggested by the auto-Ab we found in the present study, is centered on the mitochondria. Indeed, *AIFM1* is implicated in apoptosis, mitochondrial function, and redox regulation (29). In healthy cells, *AIFM1* is localized in the mitochondrial intermembrane space, where it plays a role in maintaining the mitochondrial electron transport chain and oxidative phosphorylation. It helps to sustain cellular energy metabolism by participating in redox reactions. Similarly, the human protein *SURF1* is crucial for cytochrome c oxidase (complex IV) assembly, a key component of the mitochondrial respiratory chain (30). Specifically related to PCS, research on the long-term sequelae of COVID has highlighted several mitochondrial dysfunctions, including redox imbalance, diminished capacity for oxygen extraction, and exercise intolerance (2). Additionally, deficits have been identified with mitochondrion-dependent lipid catabolism and altered fatty acid metabolism in PCS (2). Indeed, mitochondrial damage and dysfunction appear to be a hallmark of this condition (31). *DAB1*, albeit not a specific mitochondrial protein, is required in the Reelin pathway and the two proteins need to be co-expressed (32). Reelin, and therefore *DAB1* as well, is strongly associated with neuronal energetic metabolism (33). In sum, autoimmune antibody-mediated damage to *DAB1*, *AIFM1* and *SURF1* in PCS might lead to metabolic and mitochondrial dysfunction, providing a mechanistic link to reduced functional status seen in the disease.

Of note, the autoimmune damage to nervous tissue and mitochondrial damage that our results might suggest would not exclude other potential non-autoimmune mechanisms behind PCS. Indeed, multiple non-autoimmune pathophysiological processes have been proposed to underlie PCS. Viral or antigen persistence in tissues, including the gut, lymphoid organs, and brain, has been demonstrated months after acute infection and may sustain low-grade inflammation and local dysfunction (34–36). Endothelial injury, impaired fibrinolysis, and microvascular flow abnormalities have also been reported, though the significance of fibrinolysis-resistant “microclots” remains debated (37–39). Exercise intolerance and post-exertional symptom exacerbation have been linked to impaired peripheral oxygen extraction, mitochondrial dysfunction, and exercise-induced myopathy (40–42). Neuroimaging and cerebrospinal fluid studies indicate neuroinflammation, blood–brain barrier disruption, and neurovascular injury, which may contribute to cognitive symptoms (43, 44). Epstein–Barr virus reactivation has been observed in some PCS cohorts, possibly exacerbating symptoms via inflammatory and metabolic effects (45–47). Finally, persistent alterations in the gut microbiome and intestinal permeability have been described, implicating gut–immune and gut–brain axis pathways (48, 49). All these mechanisms are likely to interact; for example, viral persistence may perpetuate both immune mediated damage and endothelial injury, microvascular hypoperfusion, and mitochondrial stress, leading to clinical sequelae (34, 37, 40). In a such a complex scenario, the occurrence of auto-Abs against *DAB1*, *AIFM1*, and *SURF1* might be driven by viral persistence leading to continuous stimulation of the immune cross-reactive response and sits particularly well with mitochondrial dysfunction.

In order to better address the temporal dynamic of auto-Abs against *DAB1*, *AIFM1*, and *SURF1* the chronic phase of the disease, which the available data allowed, we tested if the measured fluorescence intensity for the IgG against the peptides from the three neuronal antigens, could predict sampling at 3 or 6 months, i.e. if the levels of the AutoAbs were substantially different between the two timepoints. Our data showed that this is not the case and we found no significant effect in this sense. Concerning clinical symptoms, the analyses on the principal components showed higher values of the second and third principal component at 6 months (**Figure 5**). So, at least persistence of the autoantibodies at 6 months after COVID can be reasonably assumed, with either persistence or slight worsening of the symptoms at 6 months.

We previously hypothesized a role of IgG against *DAB1*, *AIFM1*, and *SURF1* in cognitive impairment in COVID-19, given the well-described functions of the three proteins in neurogenesis and synaptic plasticity (11, 50–52). Nevertheless, the current exploratory study seems to challenge this idea. Specifically, no significant link was found between the autoimmune response to these proteins and cognitive performance as measured by the MoCA. Interestingly, self-reported cognitive symptoms—captured by the third principal component—were paradoxically associated with lower antibody levels. As shown in **Table 3**, cognitive complaints had a negative loading on this component, suggesting that individuals reporting such symptoms also tended to report fewer respiratory symptoms, lower disability levels (as measured by the PCFS), and reduced autoantibody levels, according to the GLMM analysis. One possible reason for the absence of a relationship between autoantibody levels and MoCA scores could be that previous research has indicated the MoCA may lack sensitivity in detecting mild to moderate cognitive impairment in post-COVID syndrome (PCS) (53). This aligns with clinical observations from consultations, where a significant proportion of patients—estimated at around 25%—do not score below the MoCA cutoff of 26 points, yet require up to twice the typical time to complete the test. As previously noted, in the context of PCS, subjective reports of cognitive difficulties often do not correlate with objective cognitive performance (27, 28). Indeed, one very recent study on self-reported health status and functioning as compared with neuropsychological quantitative assessment in PCS pattens versus controls also showed a pattern of results similar to ours. PCS patients consistently rated their own well-being and cognitive performance as lower, whereas neuropsychological batteries failed to unveil any differences, with the exception of verbal fluency (54). One additional possible explanation for our paradoxical finding is that patients with greater functional limitations and more severe respiratory symptoms may report fewer cognitive complaints, perhaps because their attention is directed more toward the physical aspects of their condition. This hypothesis warrants further investigation through targeted studies, such as comprehensive assessments of subjective health and well-being and in-depth neuropsychological evaluations, possibly focusing on language functions. We interpret the reduced power in investigating cognitive deficits in PCS as a study limitation and a stimulus to foster future specific investigations on the topic with extensive.

Moreover, note that the present exploratory study showed an association between the investigated Auto-Ab and the occurrence of PCS. A potential limitation of this study its the disparity in the PCS and control group in sex, with female sex being more represented in the PCS group, comorbidities affecting PCS patients more than fully recovering COVID-patients, and vaccination status being lower among PCS patients (**Table 1**). Although it has been shown that female sex and comorbidities are relevant risk factor for PCS and vaccination exerts a preventive effect so that such disparity is consistent with what is currently known about the disease and it is to be expected (55, 56), future research should address the contribution of this factors specifically on auto-Abs occurrence in PCS. In addition, this exploratory investigation was not designed to define diagnostic thresholds or determine precise concentrations of autoantibodies. Rather, its primary objective was to explore potential associations between the presence of these antibodies and clinical manifestations of post-COVID syndrome. To this end, we employed a linear modeling approach, using fluorescence intensity as a continuous variable to evaluate its relationship with PCS symptoms, instead of relying on arbitrary cutoffs. Importantly, the detection of autoantibodies was based not on full-length antigens, but on carefully selected synthetic epitopic regions from the human proteins *DAB1*, *AIFM1*, and *SURF1*. This deliberate focus on immunodominant fragments—previously validated in acute COVID-19—was designed to pinpoint the specific sequences most likely to trigger an autoimmune response in the context of PCS as well. However, to fully establish pathogenic relevance, future research must go beyond synthetic epitopes and examine autoantibody reactivity against the whole antigen structures.

The present exploratory study showed an association between the auto-Abs and the disease without implying a causal relationship but nevertheless offers novel insights into the mechanisms underlying reduced functional status associated with PCS and pave the way for future studies aimed at establishing a causal link between the occurrence of the auto-Abs against *DAB1*, *AIFM1*, and *SURF1* and the disease. Moreover, we could not clearly identify in the context of autoimmune damage in PCS the biological relevance of each of the three autoantigens in isolation, since we did not uncover any effect of any single peptide tested over the others. Future studies providing causal evidence for the role of the three proteins could differentiate the pathogenic relevance of each of the three proteins in the context of PCS.

Defining the pathogenic significance of this auto-Abs will have important diagnostic and therapeutic implications. First, evaluating COVID and PCS patients for the presence of auto-Abs against the epitopes outlined in this study could facilitate the identification of individuals at risk for PCS-linked higher disability. Second, these same epitopes might potentially serve, after further research replicates and confirms our findings in larger cohorts and with whole antigens and after causal evidence linking the currency of the auto-Abs here identified to PCS, as potential immunomodulatory therapeutic agents in patients who test positive for the corresponding autoantibodies.

## Data Availability

The raw data supporting the conclusions of this article will be made available by the authors, without undue reservation.
